# Progressive daily hopping exercise improves running economy in amateur runners: a randomized and controlled trial

**DOI:** 10.1038/s41598-023-30798-3

**Published:** 2023-03-13

**Authors:** Tobias Engeroff, Kristin Kalo, Ryan Merrifield, David Groneberg, Jan Wilke

**Affiliations:** 1 Division Health and Performance, Institute of Occupational, Social and Environmental Medicine, Goethe University Frankfurt, Frankfurt Am Main, Germany; 2 Department of Sports Medicine, Disease Prevention and Rehabilitation, Johannes Gutenberg University Mainz, Albert-Schweitzer-Straße 22, 55128 Mainz, Germany; 3 Department of Sports Medicine and Exercise Physiology, Institute of Sports Sciences, Goethe University Frankfurt, Frankfurt Am Main, Germany; 4 Institute of Occupational, Social and Environmental Medicine, Goethe University, Frankfurt, Frankfurt Am Main, Germany; 5 Departement of Movement Sciences, University of Klagenfurt, Klagenfurt, Austria

**Keywords:** Physiology, Musculoskeletal system

## Abstract

This study investigated the effects of a daily plyometric hopping intervention on running economy (RE) in amateur runners. In a randomized, controlled trial, thirty-four amateur runners (29 ± 7 years, 27 males) were allocated to a control or a hopping exercise group. During the six-week study, the exercise group performed 5 min of double-legged hopping exercise daily. To progressively increase loading, the number of hopping bouts (10 s each) was steadily increased while break duration between sets was decreased. Pre- and post-intervention, RE, peak oxygen uptake (VO_2peak_), and respiratory exchange ratio (RER) were measured during 4-min stages at three running speeds (10, 12, and 14 km/h). ANCOVAs with baseline values and potential cofounders as cofactors were performed to identify differences between groups. ANCOVA revealed an effect of hopping on RE at 12 km/h (df = 1; F = 4.35; *p* < 0.05; η^2^ = 0.072) and 14 km/h (df = 1; F = 6.72; *p* < 0.05; η^2^ = 0.098), but not at 10 km/h (*p* > 0.05). Exercise did not affect VO_2peak_ (*p* > 0.05), but increased RER at 12 km/h (df = 1; F = 4.26; *p* < 0.05; η^2^ = 0.059) and 14 km/h (df = 1; F = 36.73; *p* < 0.001; η^2^ = 0.520). No difference in RER was observed at 10 km/h (*p* > 0.05). Daily hopping exercise is effective in improving RE at high running speeds in amateurs and thus can be considered a feasible complementary training program.

*Clinical trial registration* German Register of Clinical Trials (DRKS00017373).

## Introduction

Competitive runners are on the constant quest for maximal performance. However, after decades of significant improvements, a trend towards more marginal changes has been observed in several disciplines including endurance running^1^. Consequently, a “marginal gains” approach, which aims at combining multiple small performance improvements in different areas to create a significant advantage, is becoming increasingly popular in amateur and professional competitive sports. Aiming at such marginal gains, amateur runners adopt complementary training approaches such as plyometrics from professional sports with the aim to maximize performance and minimize injury risk^2^.

Running performance is inherently dependent on the efficiency of locomotion, which is often referred to as running economy (RE). How efficient a human moves over the ground is not only influenced by metabolic factors, but also by the quality of movement patterns and the mechanical characteristics of the locomotor system^3^. The concept of RE takes these metabolic, neural and tissue-specific factors into account. It is defined as the oxygen uptake required per distance at a given running speed and represents one of the key parameters to quantify the ability to transform aerobic capacity into endurance running performance^3,4^. Studies in both, amateurs^5^ and elite athletes^6^, confirmed the relevance of RE as a crucial factor for endurance running performance. Therefore, strategies to improve RE are sought after by coaches, athletes, and sports scientists^7^.

Resistance training, plyometrics, and stretching represent three popular methods used to target RE^7^. A common facet of these interventions is that they all influence metabolic, biomechanical and neuromuscular efficiency^7^. Since simply replacing endurance training by an RE intervention would limit the ability to maintain or increase maximal aerobic performance, a more promising approach to maximize running performance requires the maintenance of endurance training routines to which additional methods that may improve RE are added (e.g. explosive strength exercises^3^). Popular contents of such additional training strategies include jumps, hops, or sprints and are suggested to improve muscle/tendon stiffness or to modify movement mechanics and the stretch-shorten cycle (SSC)^3^.

According to the available evidence, the Achilles tendon (AT) has been demonstrated to play a significant role in RE^8–10^. Kunimasa et al.^11^ compared a variety of anatomical characteristics of Kenyan and Japanese elite distance runners. The Kenyans, superior in running performance, exhibited higher relative AT lengths and greater AT tendon moment arms. This is of importance because both factors are positively associated with RE^9,10^. In addition to morphological features, RE is also influenced by the mechanical properties of the AT. Arampatzis et al.^8^ showed that RE is positively associated with normalized AT stiffness. Following a 14-week resistance exercise intervention, a 7%-increase in plantar flexor strength and a 16%-increase in tendon stiffness resulted in a 4% reduction of oxygen consumption^12^.

In a pioneering study, Kawakami et al.^13^ found that muscle fibers, contrary to earlier beliefs, act almost isometrically during stretch–shortening cycles (SSC). Conversely, the tendon undergoes significant length changes, storing and releasing kinetic energy. While the AT contributes more than 50% of the positive work even at low running speeds of ~ 2 m/s, this proportion increases to about 75% during sprinting^14^. In view of the accumulating evidence supporting the importance of the AT in RE, numerous studies have investigated the effectiveness of related exercise interventions. Plyometric training, often using reactive jumps, hops or, bounces when applied in the lower limb, is a popular strategy aiming to improve SSC performance. It hence seems particularly suited to trigger morphological and functional adaptation of the tendon. However, so far, only a limited number of studies examined the effect of explosiveness training interventions on RE, and only a few of them used exclusively plyometric exercises^3^. Furthermore, most available trials used one to three sessions per week, but none studied higher frequencies^3^. This is of importance because it has been shown that collagen production, which is paramount for tendon stiffness, is most effectively triggered by intermittent, progressive loading paradigms with relatively short durations and intervals^15,16^. Finally, most of the available evidence of exercise interventions and RE focusses on athletes with moderate to high-performance levels^17^. The present study therefore aimed to investigate the effects of a daily plyometric hopping intervention on RE in amateur runners. We defined amateur runners as persons who compete in sports without striving for financial reward (as opposed to professional athletes), thus do running as a hobby.

## Methods

### Study design and survey procedure

A two-arm randomized controlled trial was performed. Active amateur runners were allocated to a hopping exercise (HE) or control (CON) group. Randomization was counterbalanced and conducted using BiAS for Windows version 11.10 (Goethe University Frankfurt, Germany). The study was approved by the local ethics committee (Ethikkommission FB 05, Goethe University Frankfurt; reference number: 2018-17b) and registered at the German Register of Clinical Trials (DRKS00017373, date of registry: 03/09/2019). All participants provided written informed consent.

Healthy adults were recruited using word of mouth, printed flyers, and social media advertising. To ensure a specific fitness level and thus that participants are able to complete the running protocol, individuals had to be amateur runners with a 10 km time < 55 min and younger than 40 years of age. Exclusion criteria encompassed contraindications for engagement in physical activity (tested by means of the Physical Activity Readiness Questionnaire), severe cardiovascular, metabolic, endocrine, neural, and psychiatric diseases, unhealed orthopaedic injuries and overuse disorders (particularly with regard to the knee and ankle region), local inflammation, pregnancy, self-reported use of supplements containing stimulants and anabolic–androgenic steroids.

### Intervention

The CON and the HE groups continued their regular exercise regimes. While the CON group did not engage in an additional specific exercise intervention, the HE group completed a six-week plyometric hopping protocol. Each day, the individuals randomized to HE performed a variable amount of double-legged 10-s hopping bouts (Table 1). While total session duration (5 min) was constant, the number of sets (and with this, net training time) was increased weekly in order to ensure safe functional and mechanical adaptation. When hopping, participants were instructed to start with both feet no wider than hip width apart and to hop as high as possible with both legs, keeping the knees extended and aiming to minimize ground contact time. To ensure safe and correct execution, participants received a 1-to-1 explanation by a coach holding a bachelor’s degree in Sports Science, who additionally monitored the first three training sessions of each individual. When later exercising alone, the HE participants provided the instructor with video recordings in order to allow supervision and, if needed, correction. Moreover, participants completed a hopping diary. If at least 70% of the jumps were completed, the subjects were considered compliant.

**Table 1:** Protocol of the hopping intervention.

Week	Sets	Set duration [s]	Net hopping duration [s]	Rest between sets [s]
1	5	10	50	50
2	6	10	60	40
3	8	10	80	30
4	10	10	100	20
5	15	10	150	10
6	15	10	150	10

*s* seconds.

All participants completed a training diary, documenting weekly running activity (number of sessions, hours per session, pace) as well as other exercises in hours/ week (see Table 2) and (in case of the HE group) adherence to the hopping intervention.

**Table 2:** Description of the sample (mean values and standard deviations).

	HE group (n = 15)	CON group (n = 19)	Total (n = 34)
Sex	11♂ ♀4	16♂ ♀3	27♂ ♀7
Age [years]	29.1 (7.6)	28.2 (5.9)	28.6 (6.6)
Weight [kg]	73.8 (10.4)	78.6 (9.01)	76.5 (9.8)
Height [m]	1.78 (0.07)	1.80 (0.07)	1.80 (0.08)
Exercise [h/week]	8.0 (3.0)	8.2 (3.4)	8.1 (3.2)
Running duration [h/week]	3.3 (2.4)	2.37 (2.2)	2.8 (2.3)
Running frequency [n/week]	2.7 (1.5)	1.89 (1.3)	2.2 (1.4)
Running experience [years]	6.2 (4.7)	8.21 (5.6)	7.3 (5.3)
Running speed [km/h]	10.9 (1.8)	11.15 (1.5)	11.0 (1.6)

*n* number, *kg* kilograms, *m* meter, *h* hours, *km* kilometers.

### Measures

Before and after the intervention period, all exercise tests were performed on an electronically driven treadmill (mercury® med, h/p/cosmos sports & medical gmbh, Traunstein, Germany) without using a safety belt. To reduce intrasubject variability, the time of the day, the test equipment, as well as the type of running shoes worn were standardized for both, baseline and post-exercise tests. Participants were instructed to avoid exercise for 24 h and strenuous exercise for 48 h prior to testing. In addition, participants were asked to eat about 1.5 to 2 h before exercise testing, not to drink alcohol the day before, and not to consume any alcohol or cigarettes on the day of the test. Temperature was controlled by air conditioning and the difference in temperature between the baseline and post measurement did not exceed ± 0.5° Celsius.﻿

In accordance with Saunders et al.^18^, RE was determined by measuring submaximal oxygen uptake (VO_2_) during 4-min stages at three constant running speeds and an inclination of 0°. After a standardized warm-up of 3 min walking at 5 km/h, participants ran at 10, 12, and 14 km/h, respectively^7^. Peak oxygen uptake (VO_2peak_) was determined during a ramp protocol performed 2 min after the last submaximal running stage. For this purpose, speed was increased by 1 km/h every minute from 12 km/h and to 20 km/h, treadmill inclination was increased by 1% until volitional exhaustion^19^.

A breath-by-breath gas analyser was used to monitor gas exchange during exercise testing (Metalyzer, CORTEX Biophysik GmbH, Leipzig, Germany). Calibration of the gas as well as the flow sensor was performed according to manufacturer recommendations. Meyer et al.^20^ showed an excellent test–retest reliability for the used system (VO_2_: 0.969, VCO_2_: 0.964, V_E_ 0.953). Recorded data was stored and processed using a spiroergometry software (MetaSoft® Studio, CORTEX Biophysik GmbH, Leipzig, Germany). At the end of each running stage, the participants prepared themselves to step off the treadmill by gripping the side handles of the treadmill. Therefore, for each step, the last 10 s of VO_2_ data was cut off and RE was determined as the V̇O_2_ collected during the last valid 60 s (i. e., seconds 170 to 230) of each 4-min running stage. Respiratory exchange ratio (RER, VCO_2_/VO_2_) was calculated for these 60 s as well. During the ramp protocol, the highest individual V̇O_2_ recorded over a 30 s period within the testing time (floating mean) was defined as VO_2peak_.

### Statistical analysis

All analyses were performed using Jamovi 1.8 (The jamovi project, https://www.jamovi.org) and the significance level was set to α = 0.05. After variance homogeneity was confirmed using Levene´s Test, analyses of covariance (ANCOVA) with baseline values as a cofactor were performed to test for intervention effects (between subjects/groups) on running economy and secondary outcomes (VO_2peak_, respiratory exchange ratio). Sex was analysed as potential confounder. Further confounders (weight, frequency of regular running sessions and other exercises) were tested for between group differences using Kruskal–Wallis Tests. In case of significance, a second ANCOVA for intervention effects on running economy including baseline values for running economy and the potential confounding outcomes was carried out. For the estimates of effect sizes, eta squared (η2) was used and interpreted according to Cohen^21^: 0.01 (small effect), 0.06 (medium effect) and 0.14 (large effect).

### Ethics approval and patient consent

The study was conducted according to the ethical guidelines of the Helsinki Declaration and was approved by the local ethical review board (Ethikkommission FB 05, Goethe University Frankfurt), number: 2018-17b. All participants provided written informed consent.

## Results

**Figure 1:**
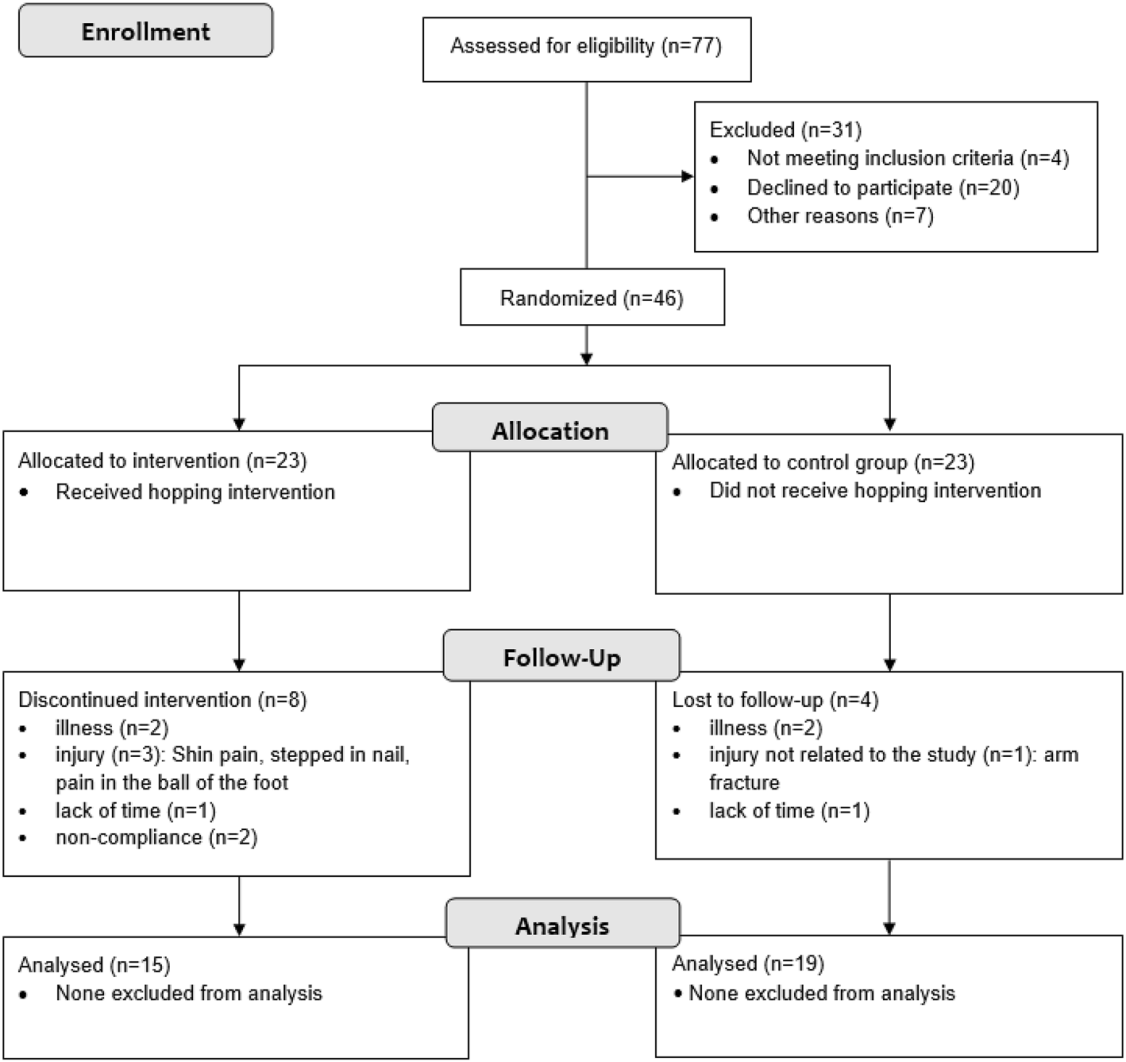
Flow chart of the study.

From n = 46 recruited individuals, a total of n = 34 adults (29 ± 7 years, 27 males) completed the study. Overall, n = 12 participants dropped out of our study. Reasons were illness (n = 4), injury (n = 4), lack of time to follow-up (n = 2), and < 70% compliant with the hopping protocol (n = 2). A detailed indication of the dropouts per group is depicted in Fig. 1.

Descriptive data of sample characteristics are presented in Table 2. None of the potential confounders including weight (df = 1; F = 0.0468; *p* = 0.830; η^2^ = 0.001), running frequency (df = 1; χ2 = 0.0305; *p* = 0.861), and general training volume (df = 1; χ2 = 0.8605; *p* = 0.354) showed significant differences between the two groups. Pre and post values of VO_2peak_, RE and RER per stage and group are presented in Table 3.

**Table 3:** Spiroergometric values (mean values and standard deviations).

	HE group (n = 15)		CON group (n = 19)		Total (n = 34)	
	Pre	Post	Pre	Post	Pre	Post
VO_2_peak [ml/min/kg]	51.4 (6.0)	51.1 (5.6)	48.26 (4.3)	50.2 (4.5)	49.7 (5.2)	50.6 (5.0)
RE (ml/min/kg), 10 km/h	35.2 (2.97)	33.9 (2.95)	34.8 (3.07)	34.8 (2.79)	35.0 (2.99)	34.4 (2.86)
RE (ml/min/kg), 12 km/h	41.1 (2.57)	40.2 (2.67)	40.4 (3.35)	41.3 (2.86)	40.7 (3.01)	40.9 (2.79)
RE (ml/min/kg), 14 km/h	47.1 (3.04)	46.1 (3.31)	45.3 (3.33)	46.9 (2.79)	46.1 (3.29)	46.5 (3.01)
RER (VCO_2_/VO_2_), 10 km/h	0.90 (0.04)	0.93 (0.05)	0.92 (0.05)	0.93 (0.05)	0.91 (0.05)	0.93 (0.05)
RER (VCO_2_/VO_2_), 12 km/h	0.95 (0.04)	0.98 (0.05)	0.97 (0.05)	0.97 (0.05)	0.96 (0.05)	0.98 (0.05)
RER (VCO_2_/VO_2_), 14 km/h	1.01 (0.06)	1.05 (0.07)	1.02 (0.05)	1.02 (0.05)	1.02 (0.06)	1.03 (0.06)

*n* number, *kg* kilograms, *h* hours, *km* kilometers, *ml* milliliters, *min* minutes, *VO*_2_ oxygen intake, *RE* running economy, *RER* respiratory exchange ratio, *VCO*_2_ carbon dioxide production.

**Figure 2:**
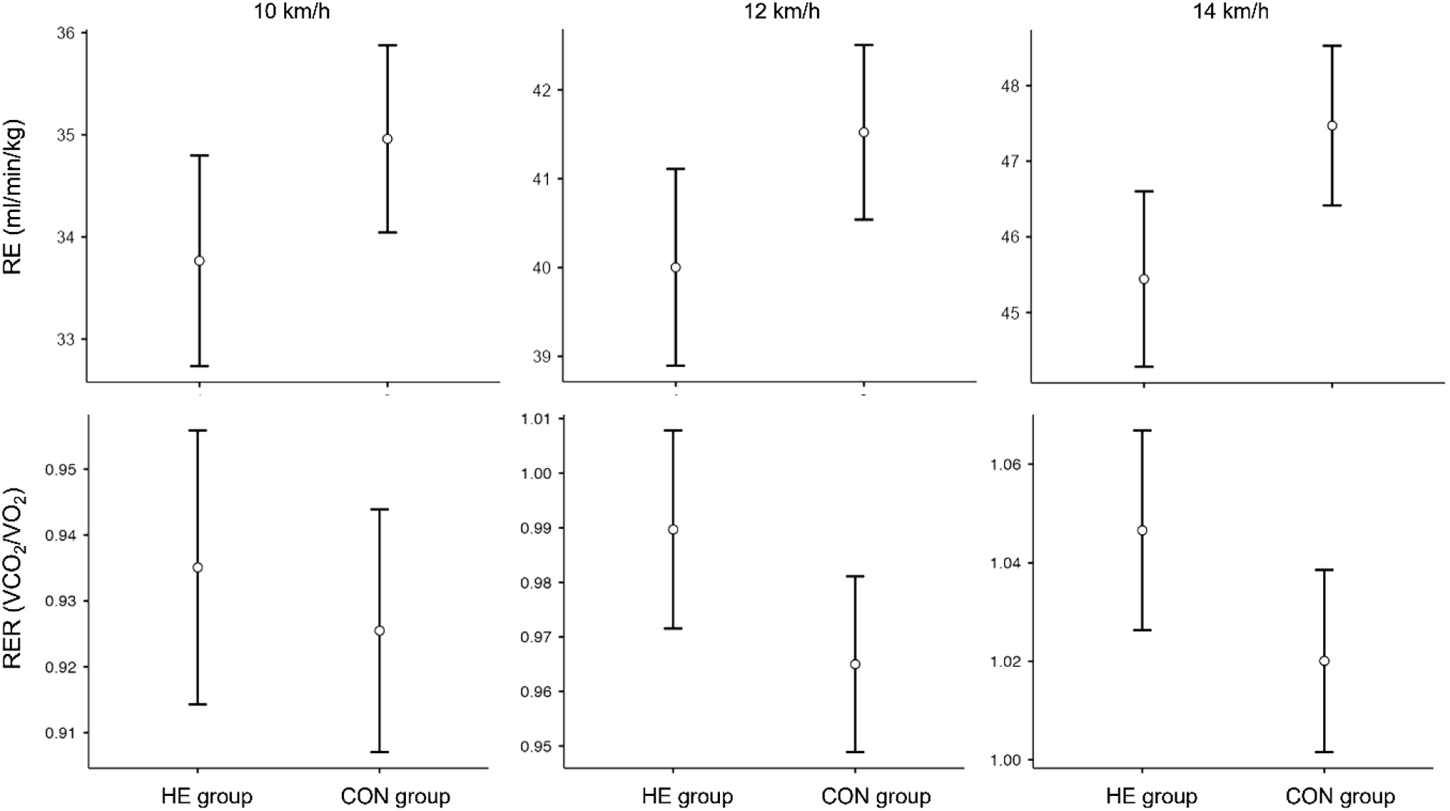
Group differences in the estimated marginal means of the post intervention values of RE and RER at three running speeds. Means (dots) and 95% confidence intervals (vertical lines) are displayed.

Levene´s test indicated variance homogeneity for primary and secondary outcomes. ANCOVA revealed that hopping significantly improves running economy at 12 km/h (df = 1; F = 4.35; *p* = 0.045; η^2^ = 0.072) and 14 km/h (df = 1; F = 6.72; *p* = 0.015; η^2^ = 0.098) running speed. In contrast, no difference between the HE and CON group was found at a low (10 km/h) running speed (df = 1; F = 3.11; *p* = 0.088; η^2^ = 0.043). HE did not lead to higher VO_2peak_ values in the HE group compared to the CON group (df = 1; F = 2.83; *p* = 0.102; η^2^ = 0.025). However, after six weeks of training, the respiratory exchange ratios during 12 km/h (df = 1; F = 4.26; *p* = 0.047; η^2^ = 0.059) and 14 km/h (df = 1; F = 36.73; *p* < 0.001; η^2^ = 0.520) running speed were significantly higher in the HE group. RER at 10 km/h running speed remained unchanged (df = 1; F = 0.490; *p* = 0.489; η^2^ = 0.011). Sex showed no impact on ANCOVA results. Figure 2 shows group differences in the estimated marginal means of the post-values for RE and RER (generated considering the pre-values) at all three running speeds.

## Discussion

The present study yielded three key findings. Firstly, six weeks of daily hopping exercise improve running economy and increase respiratory exchange ratio at higher running speeds (12 and 14 km/h) in amateur runners. Secondly, maximal aerobic capacity remains unaltered by hopping if regular running and exercise habits are maintained. Our findings are in line with earlier studies examining plyometric interventions in amateur athletes^22^ and highly trained runners^18^. They also corroborate the observation of Saunders et al.^18^ that the effects of plyometrics on running economy are more pronounced at higher running speeds. However, in contrast to the previous trials which used three weekly sessions with durations of up to 30 min as well as multiple jump exercises^18,22^, this study applied short daily bouts (net duration 5 min) consisting of one simple and easy-to-learn hopping exercise only. Our results suggest that regular endurance and concurrent plyometric training can be performed jointly by amateur runners, without complex programs and at a high frequency without leading to adverse events such as overuse injuries or pain. Finally, third, performing a progressive hopping protocol with a high exercise density seems to be safe in amateur athletes as no injury or other side effects related to the interventions were reported.

Although the present trial strengthens the evidence that plyometric exercise improves running economy, the relative contributions of factors driving the observed changes are still a matter of debate. Tendon stiffness has been shown to significantly correlate with running economy^23^. Earlier findings reported that the contribution of AT energy storage to the positive work performed during running increases with locomotion speed^14^. Our data show significant improvements in RE at 12 and 14 km/h but not at 10 km/h which would provide support for the hypothesis that tendon stiffness was one of the driving factors for improvements in RE.

Our hypothesis assumed that repeated hopping would elicit morphological and functional adaptation in the tissue, which in turn improve energy storage capacity. The daily loading paradigm was chosen based on research elucidating optimal loading paradigms for collagenous connective tissues^15,16^. For instance, Paxton et al.^15^ used engineered ligaments to study the effect of cyclic stretch on the phosphorylation of the extracellular signal-regulated kinase 1/2 (ERK1/2), an enzyme associated with collagen synthesis. Maximal ERK 1/2 levels were observed after 10 min of cyclic stretch and up to 6 h, cells were refractory to new stretching bouts. As these data suggest that repeated short loading periods may be optimal for tissue adaptation, the authors compared a daily intermittent stretch program (10 min each 6 h) to continuous tissue lengthening and confirmed the superiority of the physiology-specific paradigm. In our trial, tissue loading was of similar duration (8–14 min). Yet, as three training bouts per day with 6-h-intervals are not feasible due to nocturnal sleep, we decided for one exercise session per day. The intervention was well tolerated and just two participants report pain (shin and ball of the foot), which might be related to the intervention. Upcoming trials may therefore consider further increasing the frequency, e.g., to twice daily. Also, it has to be noted that we did not measure tendon stiffness (e.g., using an instrumented treadmill). Future studies should hence be geared to combine both, assessments of stiffness and RE.

In addition to adaptations in the tendon, metabolic factors may have caused the alterations of RE^3^. One often discussed adaptation is increased aerobic carbohydrate utilisation for oxidative phosphorylation^24^. A shift in substrate use could have led to better running economy based on a lower oxygen cost for adenosine triphosphate (ATP) synthesis if a greater share of carbohydrates is used instead of fatty acids. Since RER values in our study exceeded 1.0 at higher running speed, another explanation for decreased oxygen uptake might be an increase in anaerobic metabolism^5^. The assumption that a change in substrate use might have caused alterations in running economy is supported by an increase in respiratory exchange ratios in the hopping training group. Due to the applied method, which is based on oxygen uptake, running economy in our analysis reflects only aerobic energy metabolism. Based on our data, we thus are not able to delineate which of the aforementioned mechanisms led to the increase in RER. Furthermore, we did not control if all participants were able to maintain a metabolic steady state over a larger timeframe than 4 min during all tested running speeds and did not assess running performance using a time trial or a fixed distance. Consequently, further studies are necessary to prove, that differences in oxygen costs of running govern improvements in running performance. A third pathway for hopping induced improvements in RE could be based on changes in movement patterns^3^. Alterations in running mechanics are discussed as a possible mechanism for RE improvements induced by plyometric and explosive resistance training^7^. However, evidence on specific adaptations is scarce so far and further studies investigating running mechanics are needed. Moreover, we did not investigate the subjective acceptance and feasibility of our hopping protocol to daily (exercise) routines using structured interviews.

Our findings have implications for clinical practice and may open new avenues for future research. As indicated earlier, previous training paradigms predominantly focussed on complex and more time consuming interventions which are particularly used by professional athletes^3^. This trial provided first evidence that short daily regimes consisting of just one jump exercise are effective in improving RE in amateurs. Such regimes become more relevant as many people run and compete for fun and social aspects, but also report musculoskeletal pain caused by running^25^. In addition, the “marginal gains” approach, is becoming increasingly popular in amateur sports^2^. Therefore, hopping can be considered as short and feasible program for amateur runners to optimize performance and minimize the risk for musculoskeletal pain and injuries. Although direct comparison of a) short daily and b) longer but less frequently applied training approaches are lacking, based on current evidence, the choice could be left to individual preference. In regard to applicability, further studies should analyse if plyometric exercises can be executed in a fatigued and non-fatigued state. As an example, it is unknown whether hopping interventions performed in direct proximity to regular training (immediately before or after) would thwart the beneficial effects achieved by endurance or hopping exercises. Finally, besides endurance runners, athletes in other sports might also benefit from increased RE. Team sports such as basketball or soccer are characterized by periods of moderate intensity running which are disrupted by short bouts of high-intensity actions^26^. Consequently, RE is discussed as a relevant factor for athletic performance^26^ and training regimes might contribute to enhanced endurance and running speed in team sport athletes.

### Perspective

This study provides first evidence that 5 min of daily hopping improve RE at moderate and high running speed without compromising maximal aerobic capacity in amateur runners. This is in line with previous studies using less frequent jump exercises with higher duration^18,22^. The more frequent units nevertheless appear to be feasible and safe. However, the factors that lead to an improvement in RE are still debated. Upcoming trials should focus on comparisons of amateur and elite athletes, further increases of exercise frequency (e.g., twice daily), and the specific mechanisms underlying hopping induced RE improvements, inter alia including altered tendon stiffness and substrate utilisation.

## Data Availability

The data that support the findings of this study are available from the corresponding author upon reasonable request.
